# An impact review of a Western Australian research translation program

**DOI:** 10.1371/journal.pone.0265394

**Published:** 2022-03-31

**Authors:** Abby Mosedale, Elizabeth Geelhoed, Yvonne Zurynski, Suzanne Robinson, Kevin Chai, Delia Hendrie

**Affiliations:** 1 School of Population Health, Health Economics and Data Analytics, Curtin University, Perth, Western Australia, Australia; 2 School of Allied Health, University of Western Australia, Perth, Western Australia, Australia; 3 Telethon Kids Institute, Perth, Western Australia, Australia; 4 Australian Institute of Health and Innovation, Macquarie University, Sydney, New South Wales, Australia; 5 Curtin Institute for Computation, Perth, Western Australia, Australia; Illawarra Shoalhaven Local Health District, AUSTRALIA

## Abstract

The translation gap between knowledge production and implementation into clinical practice and policy is an ongoing challenge facing researchers, funders, clinicians and policy makers globally. Research generated close to practice and in collaboration with end users is an approach that is recognised as an effective strategy to facilitate an improvement in the relevance and use of health research as well as building research capacity amongst end users. The Research Translation Projects (RTP) program funded by the Western Australian (WA) Department of Health facilitates clinical and academic collaboration through competitive funding of short-term research projects. Its aim is to improve healthcare practice while also finding efficiencies that can be delivered to the WA health system. A mixed methods approach was adopted to evaluate the research impact of the RTP program, at completion of the two-year funding period, across a range of impact domains through the adaptation and application of the Canadian Academy of Health Sciences’ (CAHS) framework for research impact. In addition, further analysis was undertaken to address specific objectives of the RTP program more closely, in particular research capacity building and collaboration and health system Inefficiencies targeted by the program. Social network analysis was applied to assess the extent and growth of collaboration across WA health organisations over time. Results indicated that the ‘bottom up’ approach to research translation has triggered modest, yet positive outcomes across impact domains including advancing knowledge, collaboration and capacity building as well as contributing to changes in policy and practice. Additionally, the projects identified opportunities by which inefficiencies in the health system can be addressed. Further work is required to better understand the pathways by which short-term outcomes can be translated into more long-term impacts and the mechanisms that trigger this process.

## Background

The production of quality evidence to inform policy and practice in healthcare requires significant investment. However, implementation and translation of healthcare research into policy and practice is an ongoing challenge facing researchers, funders, clinicians and policy makers globally [1]. This translation gap between knowledge production and implementation into practice is widely recognised in the health research community. As a consequence of this gap, the health system does not meet its potential to provide patients with advances in technology and cost-effective healthcare programs and interventions. As a result, the return on the original investment in research could be brought into question [1, 2].

Research translation is a complex process, with many factors and conceptualisations to be considered. The ‘bench-to-bedside’ process of research translation whereby applying knowledge from basic sciences to produce new medicines, devices and treatment options for patients focuses on the interface between basic science and clinical medicine [3]. From a public health and health services research perspective, the emphasis is on the healthcare delivery systems and improving health through the translation of research into policy as well as practice [4]. In addition to the conceptualisation of research translation, terminology varies throughout the literature to include knowledge translation, evidence-based practice and knowledge to action [4]. Despite the semantics, all acknowledge a gap between the knowledge from research and its application to, policy and practice, and treatment options, and the importance of closing this gap. In addition, regardless of the perspective taken, there is agreement that this process is dynamic, involving interaction between researchers and end users, as well as the context of implementation [5]. One such complex interaction identified in the literature is the ‘top down’ process of research translation whereby researchers introduce pre-conceived practices or interventions to a system of care and ask clinicians to assist in its implementation, requiring adaptation to the local context and leading to poorly sustained interventions [6, 7]. The top-down approach comes with an expectation that management and resource structures are in place to enable clinicians to implement the change. A ‘bottom up’ approach may be the response needed to address the research translation gap, whereby research is generated close to practice in collaboration with end users. Collaborative research from a healthcare perspective, which is the organised and deliberate interactions and processes between researchers and end users (clinicians, decision makers and patients) is recognised as an effective strategy for increased relevance and use of health research as well as building research capacity amongst end users [8–11]. To facilitate this approach to research translation, research funders are enabling translational research projects and programs through unique funding opportunities. On a large scale, such funding programs have been seen in Canada, the United Kingdom and Australia [12].

A smaller scale example is the Research Translation Project (RTP) program, funded by the Western Australia Department of Health (WA Health). The RTP program is a competitive program, funding short-term research projects. Its aim is to improve healthcare practice and/or policy by investigating potential efficiencies that can be delivered to WA Health while maintaining and/or improving patient outcomes [13]. The RTP program encourages applications in the areas of clinical research, health services research, and public health research [13]. Projects must address relevant contemporary challenges faced by the publicly funded health system in WA by undertaking new research, proof-of-concept and/or pilot studies or local application and evaluation of research that has been applied elsewhere. Collaboration between disciplines and institutions is a key priority for successful funding. In addition, proposals that are likely to become the groundwork for further implementation, commercialisation or research grant applications to national funding bodies such as the National Health and Medical Research Council (NHMRC) are also strongly encouraged.

While funding organisations are increasingly focused on creating opportunities for research translation in the form of funding programs, less attention has been paid to the evaluation of such programs [14]. McLean, Graham [14] highlight the lack of evaluation of knowledge translation programs at the funding level and call for evaluation that identifies what works, for what type of funder and why.

While internal evaluation and monitoring of the RTP program has been undertaken, a more formal evaluation has the capacity to better inform ongoing investment. The measurement and assessment of returns on research investment is high on the policy agenda in Australia and across the globe [15]. Additional to accountability upwards to funders and downwards to users, the evaluation of research impact can also contribute to the development and strengthening of research dissemination and translation, inform ongoing funding allocation to bring about desired impact as well as manage and monitor the performance of research programs [16, 17]. The aim of this study is to evaluate the research impact of the RTP program across a range of impact domains consistent with the programs focus. Specific objectives of the study are to: (i) assess the research impact of research translation projects at the completion of funding against an adapted version of an existing evaluation framework, (ii) undertake social network analysis of collaboration across the WA health system as a result of the RTP program and (iii) identify sources of inefficiency addressed by projects and the proposed decision-making informed by the project outcomes.

## Methods

A mixed methods approach was adopted to evaluate the RTP program based on document analysis. Research impact of the program was assessed along the spectrum of research translation through the adaptation of an existing framework [18]. In addition, further analysis was undertaken to address the objectives of the RTP program more closely, specifically with regard to research capacity building and collaboration as desired outcomes of the RTP program as well as areas of inefficiency targeted by projects funded under the RTP program.

### Selection of the evaluation framework

Driven by a policy agenda that demands accountability, reduced waste and a focus on sustainable health systems, research impact has become a topical issue and, as such, there is growing interest in measurement internationally. We drew on the literature to identify a suitable conceptual framework to support the RTP program evaluation. A number of authors have attempted to summarise the use of research evaluation frameworks and tools presented in the literature through comparative studies and systematic reviews, including the “real world” impact of research, rather than just that which is applicable to academia [1, 16, 19, 20]. Cruz Rivera, Kyte [16] reported on twenty-four unique methodological frameworks for assessing health research impact. In recent reviews, both Cruz Rivera, Kyte [16] and Raftery, Hanney [19] have identified the Payback Framework as the most widely applied model for the evaluation of funded health research programs, a finding that is consistent with a review, conducted by Hanney, Buxton [21]. The Payback Framework developed by the Health Economics Research Group at Brunel University was originally designed to assess the return on investment from health service research [22]. The concept of “payback” as the foundation of the work of Buxton and Hanney [22] focuses on the benefit of research across a number of domains, such as knowledge, future research, research use and political and societal benefits. In recent years, the Payback Framework has been applied and adapted in the development of other methodological frameworks [16, 19].

The framework chosen here for the assessment of research impact of the RTP program is the Canadian Academy of Health Sciences’ (CAHS) framework [18], an adaption of the Payback Framework. The CAHS framework was selected because it provides a menu of defined and published preferred indicators and metrics for each category and subcategory of the framework; a unique advantage not provided by other frameworks. We chose indicators that are generic and could be applied across the four pillars of research outlined in the CAHS, namely biomedical research, applied clinical research, health services and policy research and population and public health research [18]. The adaptability of the CAHS framework across these four pillars of research is important given the heterogeneity of the research projects funded through the RTP program. Additionally, this evaluation of the RTP program is over a relatively short timeframe, with projects being assessed up to the end of a two-year funding period. The CAHS framework considers research impact along a pathway of translation, which allows outcomes that are feasible to assess in the short term to be evaluated. Finally, the CAHS framework was selected based on its previous use within the RTP program, for internal evaluations as well as in program documentation.

The CAHS framework is designed to capture health research impact across five main categories: 1) advancing knowledge, 2) building capacity 3) informing decision‐making, 4) health impacts, and 5) broad socio‐economic impacts. Given the short timeframe for the evaluation, research projects were only assessed across the first three categories. Research impact in categories 4 and 5 are unlikely to be achieved at the completion of a two-year project. As instructed by the framework developers, the set of indicators and metrics chosen for this evaluation were based on their ‘feasibility’ and ‘attractiveness’, primarily on account of data availability, timeliness, and attribution.

A key goal of the RTP program is to facilitate clinical and academic collaboration. Literature suggests that a benefit of clinical and academic collaboration is the development of research capacity amongst the clinical workforce, which has been argued to be fundamental to closing the evidence–practice gap [23]. In addition to using the CAHS framework for measuring research impact, the principles of capacity building proposed by Cooke and colleagues [23–25] were applied to assess the contribution of the RTP program to research capacity building across the WA health system. These principles are 1) skill development 2) developing research ‘close to practice’ 3) building linkages and partnerships 4) appropriate dissemination 5) continuity and sustainability and 6) establishing infrastructure.

In addition to facilitating clinical and academic collaboration the RTP program also seeks to improve efficiencies in the WA health system. Due to the analysis being undertaken in the short term, a framework to capture the types of system inefficiencies at the centre of each project was developed based on the work of Ravaghi, Afshari [26] on hospital inefficiencies and the World Health Organization on technical inefficiencies relating to health system inputs [27]. Four broad types of inefficiencies were identified: 1) inefficient use of procedures, investigation and equipment, 2) irrational use and supply of drugs and blood products, 3) inappropriate or costly workforce mix and 4) inappropriate hospital admissions and length of stay and suboptimal quality of care.

### Data sources and extraction

The primary source of data was archival documents of the RTP program including application guidelines, grant applications and completion reports from researchers. Data extraction was undertaken using a systematic examination of completion reports and corresponding grant applications for RTP projects funded between 2011 and 2016. Each grant recipient is required to submit a completion report to provide an account of objectives achieved, whether or not the program/intervention was found to provide efficiencies to the health system and whether the funding had led to other benefits (such as changes in culture, capacity, new collaborations etc.). Completion reports also provide information regarding research outputs such as publications and presentations resulting from the funded research. Given the two-year funding period of the RTP program, the selection of projects funded between 2011 and 2016 provided sufficient time for project completion and submission of final completion reports, with an allowance of time for slippage. Projects were included in the review only where the final reports and corresponding applications were available. For the assessment of collaboration, applications for successfully funded projects were included beyond 2016 up until 2019. Given that the applications and reports are completed by the research teams, the data they contain is self-reported and the data quality is limited to the level to which the applications and reports are completed.

### Data analysis

Several approaches were applied to analyse data extracted from the RTP documents, depending on the indicators and metrics used. These included content and thematic analysis and social network analysis. In the first instance, a content analysis against indicators and metrics selected from the CAHS research impact framework was undertaken for descriptive quantitative analysis (i.e. counts). Table 1 identifies the specific indicators and metrics extracted from each document as part of the content analysis.

**Table 1:** Research impact indicators.

Advancing knowledge	Number of peer-reviewed publications
	Number of peer reviewed publications in high impact (Q1) journals^1^
	Number of reporting publications in draft (Y/N)
	Number of other publications (e.g. grey literature, reports educational material)
	Number of contributions to conferences or symposiums
	Media appearance (e.g. radio, television, web-page feature) (Y/N)
capacity building	Additional funding (applied or secured) (Y/N)
	Number of higher degree students
Informing decision making	Changes/implementation of new local practice guidelines/policy (ward/unit/setting where project was directly implemented) (Y/N)
	Changes/implementation of new practice guidelines /policy beyond local setting (Y/N)
	Actions to inform/engage policy makers (e.g. briefing policy makers) (Y/N)

^1^Q1 denotes the top 25% of impact factor distribution in that journals subject category [28].

Guided by Sarre and Cooke’s principles of research capacity building [25], further analysis of the collaboration between clinicians and academics was undertaken using social network analysis (SNA) and thematic analysis [25].

The SNA was conducted at the organisation level, identifying the organisations represented within each research team between 2011 and 2019. Organisations represented on each successfully funded project team were coded into categories (Table 2). Nodes in the network graph are used to represent the organisations while the connections (edges) are weighted (i.e. drawn thicker) based on the number of project collaborations. The data analysis and processing was developed using Python and NetworkX (https://networkx.org/), a module for network analysis, in addition to developing a Web application using a JavaScript library named vis.js (https://visjs.org/) for building interactive network visualisations.

**Table 2:** Categories of participating organisation.

Clinical service organisations	Academic organisations	Consumer organisations
WA health service provider	Research institute	Consumer organisation
WA primary care	NFP condition-based	
WA rural hospital	WA university	
WA metro hospital	Non-WA university	
WA health organisation	Overseas university	
Non-WA health organisation	Private consulting	
Non-WA health service provider		
Non-WA hospital		
Non-WA primary care		
Other health service		
Overseas health service provider		
Other health service		
Overseas hospital		
Private hospital		

Deductive thematic analysis was also undertaken to illustrate the contribution of the RTP program to capacity building. Direct quotes reflecting the principles of research capacity building developed by Cooke [24] were identified in RTP final reports and coded based on these principles. Finally, sources of inefficiency and proposed solutions were identified from information presented in the RTP final reports and coded based on the adapted framework developed from the work of Ravaghi, Afshari [26] and the World Health Organization [27].

Curtin University Human Research Ethics Committee (HREC) has approved this study. Approval number: HRE2020-0464.

## Results

### Research impact

**Fig 1:**
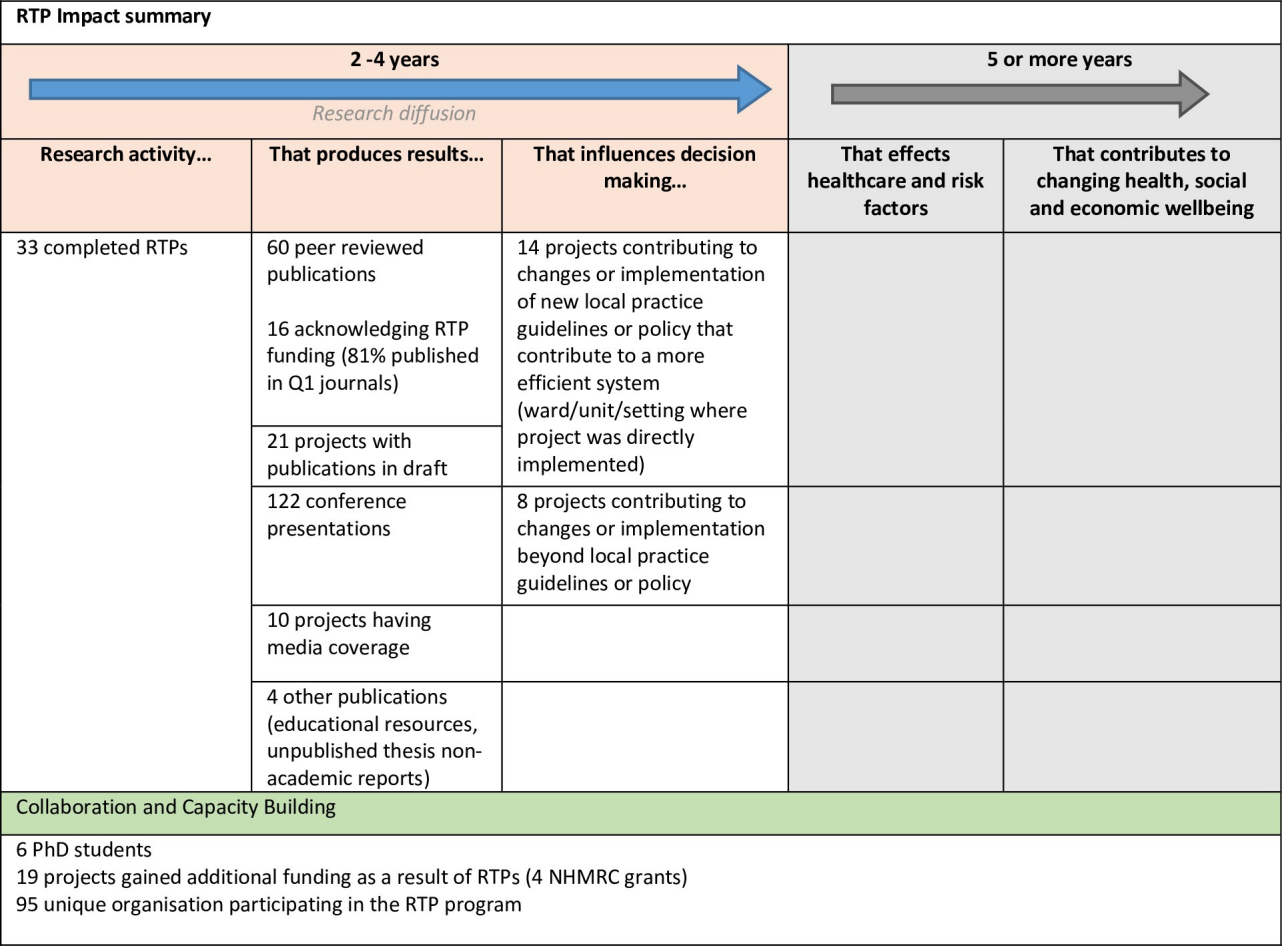
Summary of research impact of the RTP program. (Adapted from https://www.cahs-acss.ca/wp-content/uploads/2011/09/ROI_FullReport.pdf).

Of the 59 projects funded between 2011 and 2016, the required documentation was available for 33 projects for which the research focus spanned across a spectrum of acute hospital-based research to community care, general practice as well as diagnostics and clinical education From a broader point of view the RTPs by nature of the grant guidelines are focused on quality improvement issues. In addition the cost savings perspective means that the research projects are largely quantitative, pre-post or before after studies. Project funding was for a term of two years with a maximum amount of $270 000. Fig 1 provides a summary of the short-term research impacts in terms of the traditional impact measures. The longer-term impacts, beyond 5 years, such as the effects on healthcare and risk factors and contributions to changing health, social and economic wellbeing were beyond the scope of this study. In relation to Advancing knowledge, 60 peer- reviewed publications were reported as having been published as a result of RTP funding, however, upon citation analysis only 16 publications acknowledged this funding. Of these 16, 81% (n = 13) were published in high impact (Q1) journals [28]. Twenty-one projects reported publications in draft at the time of completion. There were 122 conference presentations resulting from the 33 projects, and 10 projects reported media coverage. Other publications noted in the final reports included educational resources and unpublished theses.

Within the category of capacity building, content analysis revealed that across the traditional measure of capacity building, of the 33 projects, six PhD candidates used the projects for research contributing to their doctoral award. Nineteen projects gained additional research funding leveraged off the initial RTP funding, four of which were NHMRC grants.

Finally, in relation to influencing decision making, 14 projects reported a contribution to the implementation of new local practice guidelines or policy within the setting where the project was undertaken. Eight projects reported making contributions either to changes to policy and guidelines beyond the local setting.

### Research capacity building and collaboration

Both thematic and social network analysis was undertaken to provide a deeper understanding of the extent of collaboration and research capacity development as a result of the RTP program.

#### Thematic analysis

The review of narratives on research capacity developed as a result of the RTP program reflected capacity building in line with the six principles proposed by Cook (2005) (Table 3). Additionally, improved collaboration was a noticeable outcome of the RTP program as reported by investigators in terms of both clinical collaboration and research collaboration.

**Table 3:** Illustrative quotes of capacity building resulting from the RTP program.

Cooke framework for Research Capacity Building (2005)	Excerpt from final report narrative
***Principle 1*. *Skill development***	*“Personnel capacity building has resulted from the project*, *everyone involved has had the opportunity to increase their research skills*, *in particular*.* *.* *. *the principle people involved in the project*. *This project has required extensive data collection and analysis and has provided plenty of opportunity to gain experience writing for publication and preparing presentations*.*"*
*Research capacity is built by developing appropriate skills and confidence*, *through training and creating opportunity to apply skills*	
	*“This translational study has also increased the interest and involvement of clinical staff (anaesthetists*, *registrars*, *anaesthetic technicians*, *surgeons and nurses) in research and resulted in various other additional studies with clinical collaborators*.*"*
	*“The research has supported the professional development of various members of our team in different ways including helping to build new collaborations across disciplines*, *fostering the training and development of research students and staff*, *and providing the opportunity to test in practice ideas from theory*.*"*
***Principle 2*. *Developing research ‘close to practice’***	*“This study highlighted the difficulty in recruiting patients in an imaging department where staff do not treat or look after patients for a long period of time*. *It demonstrates the importance in research of a strong collaboration between motivated treating physicians who have an established longer-term relationship with the patients and the imaging provider for an efficient recruitment”*
*Research capacity building should support research ‘close to practice’ in order for it to be useful*	
***Principle 3*. *Linkages*, *partnerships*** *and collaborations enhance research capacity building*	*“Funding for this study has provided the platform for our team to grow and generate other collaborative projects with institutions and research groups…"*
	*“Through the reporting of our initial results we gained international presence which led to collaborations with ENT units in Japan*, *South Korea*, *New Zealand and Germany with the aim of undertaking an international multicentre study*.*”*
	*“This project has brought together researchers*, *consumers and practitioners in the areas of medicine*, *law*, *psychology and aged care*.*"*
	*“The project team has also begun to establish a collaborative relationship…*. *the collaboration will seek to establish pathways between community managed organisations and mental health units/emergency departments for consumers…”*
	*"The project created the rare opportunity for specialist anaesthetists and GPs to interact directly*, *increasing both groups awareness of the need to share the responsibility for preparing the patient for elective surgery*.*"*
***Principle 4*. *Appropriate dissemination***	*16 peer review papers acknowledging RTP funding (81% published in high impact journals)*
*Research capacity building should ensure appropriate dissemination to maximise impact*	*21 projects with publications in draft*
	*122 conference presentations*
	*10 projects having media coverage*
	*4 other publications (educational resources*, *unpublished thesis non-academic reports)*
***Principle 5*. *Continuity and sustainability***	*“…the project has helped us generate more ideas for research*, *with the aim to improve patient care*. *For the hospital and health system at large*, *implications may include further investment into quality*, *evidence-based research to inform and enhance practice*, *whether this be at clinical*, *financial or operational levels*.*"*
*Research capacity building should include elements of continuity and sustainability*	
	*“"We have subsequently applied to conduct a large RCT…*. *This has been submitted as a project grant in the latest round of NHMRC funding applications and received extremely positive feedback*. *We hope to begin this trial in 2018*.*"*
	*“"This grant has significantly assisted with research capacity building*. *Most notably*, *the $200000 of funding and the commencement of the research was able to be successfully integrated into a successful NHMRC Partnership Grant Application…with $1*,*488*,*315*.*26 awarded "*
***Principle 6*. *Infrastructure***	*“The research has enabled us to validate our locally developed nomogram which we see as a unique improvement on the current means of assessing discharge risk…We used the opportunity of the research to develop a…policy/escalation plan for encountering a deteriorating patient or unexpected home visit problems*, *that is being integrated into clinical practice*.*”*
*Appropriate infrastructures enhance research capacity building*	
	*“The funding provided for this project enabled the employment of a data manager and a nurse who helped with screening a very large number of potential patients*, *recruitment*, *scanning and data collection…The data manager and nurse employed for this project will remain in their position and use their skills on a number of new funded research studies*:*”*

#### Social network analysis

**Fig 2:**
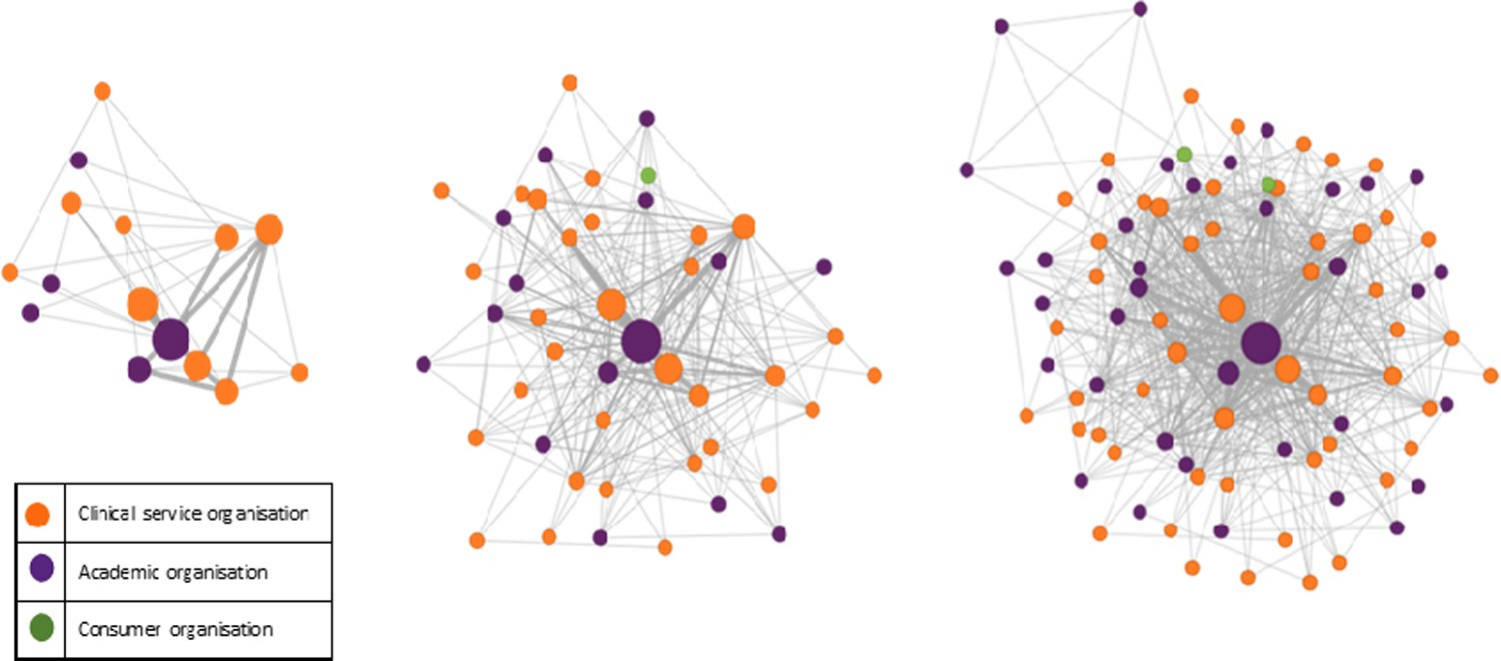
Research collaboration by type of organisation: Clinical services, academic or consumer.

The extent of the collaboration network generated by the RTP program across the WA health system was depicted using SNA. Fig 2 represents the cumulative growth of the RTP collaboration network at time intervals 2011, 2015 and 2019. Each node on the diagram represents an organisation that has participated in the RTP program as part of a project team and each line represents a collaborative relationship. The size of the node is indicative of the number of projects related to each organisation.

**Fig 3:**
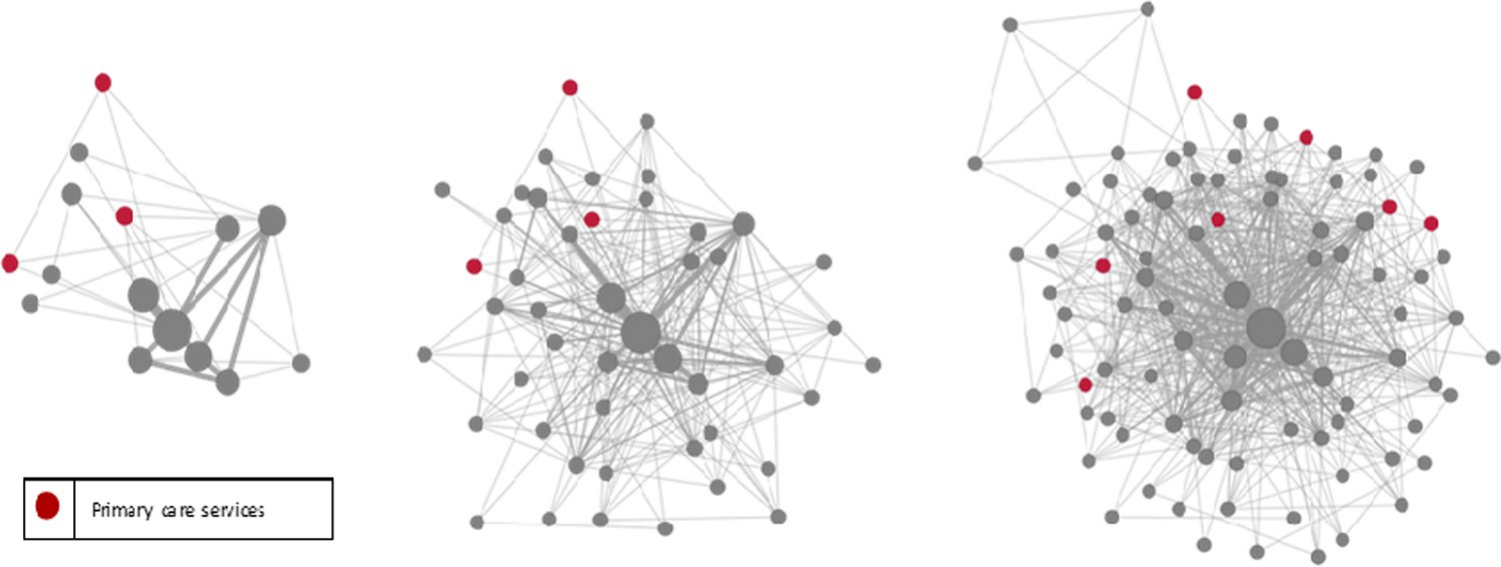
Research collaboration by type of organisation: Primary care or other.

Over this time period, 95 unique organisations were represented on funded projects. There was cumulative growth in the RTP collaborative network over time with an increasing number of organisations and collaborative relationships added in 2015 and 2019 compared with 2011, (Fig 2). To illustrate the collaboration between academics and service providers, academic organisations are represented as purple nodes and clinical service organisations as orange nodes. For details of organisations included in each group, refer to Table 2. It is important to note that two nodes, identified in green represent consumers or consumer representative organisation. In addition to an overall growth in the collaborative network, Fig 3 distinguishes organisations with a special interest in primary care (red nodes), highlighting their increased involvement (although small) of these organisations in the RTP network over time.

#### Sources of inefficiency

An additional feature of the RTP program is its focus on efficiencies and generating cost saving. Report narratives provided by investigators suggested that, as a result of participating in the RTP program, clinicians and researchers developed a sense of resource awareness and an appreciation for undertaking economic evaluation as part of intervention research.

*"Being involved in this study has provided internal staff with experience in clinical research and the value of analysis of cost effectiveness and considering new models of care to maximise the health dollar"*

*“The project has given researchers at the… a better understanding of health economics…”*

Of the 33 projects assessed, 67% (n = 22) attempted to address service delivery inefficiencies. The majority of these primarily aimed to reduce inpatient hospital costs related with inappropriate hospital admissions and length of stay and suboptimal quality of care (Table 4). Strategies employed to address these inefficiencies included improved coordination of care, shifting care from inpatient to community or home-based care and implementation of care pathways for inpatient admissions. Six projects (n = 6) attempted to address inefficiencies in relation to tests and procedures, proposing new test or procedure technology that could deliver the same or better outcomes at a lower cost. Three projects focused on inefficiencies relating to the irrational use and supply of drugs or blood products. Examples included the implementation of new guidelines to improve dispensary efficiency and effectiveness, and the trial of an alternative, more cost-effective therapy. Finally, workforce substitution from medical staff to allied health staff was the focus of two projects (n = 2) to address inefficiencies related to an inappropriate or costly workforce mix.

**Table 4:** Sources of inefficiency addressed by research translation projects.

Type of inefficiency	Common sources of inefficiency	Proposed actions from RTP	RTP projects addressing source of inefficiency
**Service delivery**	Inappropriate ALS*, unnecessary admissions or unnecessary referrals to specialists due to historical and inappropriate practice. Inappropriate service availability. Insufficient guidelines and treatment plans during admission	Improving coordination of care and care management processes; Shifting care from hospital inpatient to community/home setting; Implementation of follow up and screening services to reduce hospital admissions and LOS; Implementation of care pathways for inpatient admissions; intervention to improve provider communication and collaboration; patient education; clinician education; implementation of adjunct therapy to reduce LOS	n = 22
Inappropriate hospital admissions and length of stay and suboptimal quality of care			
**Tests and procedures**	Misuse or inappropriate use of technology in patient treatment and diagnosis like imaging and lab services due to lack of adopted evidenced-based guidelines and technologies and/or lack of knowledge and skills of health professional.	Implementation of new technology to reduce cost of test procedure.	n = 6
*Inefficient use of procedures*, *investigations and equipment*			
**Medicines and blood products**	Limited knowledge about lack of therapeutic effect; inadequate regulatory frameworks. Lack of knowledge about cheaper alternative.	Implementation of new guidelines, improving dispensary efficiency and effectiveness, trial of alternative therapy	n = 3
*Irrational use and supply of drugs*. *Inefficient use of blood products*			
**Workforce**	Suboptimal use of workforce capabilities, including those of physicians, nurses, paramedics, and allied health.	Role substitution to allied health.	n = 2
*Inappropriate or costly workforce mix*			
**Total projects**			n = 33

## Discussion

This study reports on the assessment of the RTP program against established research impact indicators including advancing knowledge, building capacity and informing decision making in line with the short-term domains of the CAHS framework, leaving the long term domains for future investigation. While common indicators of advancing knowledge and capacity building have been widely used and reported in the literature (e.g. publication count, additional funding etc.), we have applied more novel methods such as SNA to evaluate capacity building and collaboration in greater detail in addition to the traditional approaches.

Based on the findings from this study, the RTP program has made some notable contributions to a range of established research impact categories. Despite over-reporting by investigators, evidenced by the 60 peer review publications reported but only 16 explicitly attributed to the RTP grant funding, the program has demonstrated research output in the form of peer reviewed publications, conference presentations, media appearances and other publications such as educational resources, unpublished thesis and non-academic reports. It is recognised that counting publications is a simple way to measure research output and is a limited indicator of impact without an accepted benchmark for comparison [18]. Given the short time frame over which projects were assessed it was unrealistic to undertake a citation analysis at the completion of funding. In addition to the number of publications, the quality of publication output was of a high standard, as evidenced by the majority of publications appearing in high impact journals [28]. While the RTP program has demonstrated achievement across the 3 short term domains of the CHAS framework the research translation projects were not without issues. Several projects identified problems with recruitment, ability to access data, changes to the practice setting, changes to funding of services and movement of staff away from the study area, all of which impacted on the completion and quality of research.

A key aim of the RTP program is to integrate health professionals more effectively into clinical research activity that addresses clinical needs by providing practical solutions on the frontline of care. There was evidence within the project completion reports that the RTP program led to changes to policy or implementation of new practice guidelines within the local setting of the intervention and in some cases beyond the that setting, which is an illustration of its ability to contribute to evidence-based policy and practice. However, the extent of uptake of such practice guidelines and policy and the sustainability of such changes are not well understood.

Beyond the traditional measures of research output, the RTP program, with relatively modest funds, appears to have made some contribution to developing research capacity and collaboration across the WA health system. The social network analysis illustrates the breadth of organisations who are collaborating across the WA health system via the RTP program. While there is currently limited involvement of consumer groups and primary care, this appears to be growing over time which is an important finding given the commitment of the WA department of health to collaborate and engage more meaningfully with consumers and primary care through research, evaluation and health service improvement as outlined in the sustainable health review [29]. Increased collaboration across acute and academic organisations with primary care is also beneficial as we attempt to move away from hospital based care to preventative care.

This study has highlighted research capacity building as an added value of undertaking collaborative research. While collaboration is inevitable because of the nature of the projects, research capacity building can be seen as a by-product of the opportunity provided throughout the RTP program to undertake collaborative research. This was evidenced by specific reference to research capacity building principles in the report narratives. In addition, indicators for capacity building from the CAHS framework were also reported on, with the program contributing to a number of higher degrees and supporting the procurement of additional funding. Research capacity building is considered fundamental to closing the evidence–practice gap [23] and advancing both the individual and organisation’s ability to conduct, translate and sustain higher quality research and practice [30–32]. The development of skills and research knowledge as the first principle of research capacity building was a powerful message that came across throughout the majority of the reports assessed. The importance of programs such as the RTP program is therefore accentuated by the growing concerns for the research capacity of the clinical workforce and the competitiveness of clinician-researchers when it comes to securing funding for quality research [33–35].

In addition to increasing research skills and knowledge, collaboration and linkage was a notable benefit reported by investigators as a result of undertaking an RTP. The SNA effectively demonstrated the growth in the RTP collaborative network over time and the balance of collaboration between health service organisations and academic institutions. The notion of building capacity in research and facilitating collaboration is dependent on funding and support opportunities from funding like the RTP program. Given the benefits of clinician engagement in research outlined in this paper, initiatives to develop and facilitate such opportunities should be supported.

The evaluation focus was over a short time period, which meant only the research outputs and impacts that were present at the end of the funding period could be captured and long term impacts which are subject to significant time lags such as health impacts, and broad economic and social impacts where not able to be assessed [16]. While the broad economic impacts were unable to be assessed, we were able to identify the specific sources of inefficiencies that each of the RTPs where addressing to reduce waste in the system. The largest source of inefficiency to have been addressed was in service delivery where inappropriate hospital admissions, length of stay and suboptimal quality of care drive up healthcare costs. Other opportunities that were adopted to promote greater efficiency and a more sustainable heath system were the inefficient use of procedures, investigations and equipment, the irrational use and supply of medicines and blood products as well as inappropriate workforce mix. While the detail of economic analysis presented in the final reports made it difficult to estimate that tangible cost savings had been made, eligibility for RTP funding requires clinicians and researchers to actively seek more efficient ways of delivering healthcare across a range of inefficient practices without compromising health outcomes.

While this study has provided important insights into the contribution of the RTP program to indicators of research impact, capacity building and identifying opportunities for efficiency gains in the WA health system, insight into how research translation projects such as those funded by the RTP program achieve these outcomes has not been explored. Future research on the RTP program is planned, delving into the mechanisms that lead to positive outcomes such as research translation and its longer-term contribution to overall health system sustainability.

### Limitations

The completeness of the final reports submitted by investigators varied greatly and, in many cases, completion reports were poorly done. With only 33 out of 59 projects able to be assessed, this in itself reflects waste to the system as there might have been important findings to improve efficiency that were missed. Incomplete reporting also potentially introduces a degree of bias into the study’s results. The self-reporting nature of the data collection may also be a limitation with the potential for self-reporting bias and overstating positive outcomes of the research as well as a bias towards completion with only those who felt they were successful bothering to complete the final report The short-term nature of the evaluation is a further limitation to capturing the outputs and impacts that may have arisen over time. It is widely acknowledged that research impacts on health and broader societal and economic outcomes is associated with significant time lags. There is therefore a need for further evaluation beyond the two-year funding period to better understand the extent of theses outcomes and impacts of the RTP program.

## Conclusion

The RTP program has contributed to common measures of research impact such as advancing knowledge, building research capacity and collaboration and, to a lesser extent, contributing to few yet worthy changes to policy and practice. These outcomes have been achieved through clinician-initiated projects, in collaboration with academic researchers, and implemented within local health organisations in response to an opportunity to initiate change with a potential to generate efficiencies. This approach to initiating change is consistent with one of the enduring strategies put forward by the recent Western Australia Sustainable Health Review, which reiterates the notion of partnerships with clinicians, consumers and a wide range of partners being needed to develop, test and spread initiatives that deliver better patient care, and are vital to achieving a more sustainable health system [29].

While the research presented here highlights the short term research outputs and contributions to research capacity building and collaboration, it does not explore how these outcomes were achieved or the mechanisms which may contribute to the spread and uptake of research and innovations across the system in the longer term. In addition, a lens that looks beyond the two-year funding period would provide insight into research outcomes that are subject to time lags, such as changes in policy and practice and economic impacts at a system or societal level. Further research is required to better understand the mechanisms triggered by the RTP program to facilitate the spread and uptake of research innovation across the system over time. Understanding the *‘how’* will enable the recommendation made by the Sustainable Health Review to be implemented more effectively to achieve a more sustainable health system.
